# An Immature Traumatic Teeth Management With Apical Pathology Using the Novel Biodentine™ Obturation: A Case Report

**DOI:** 10.7759/cureus.20818

**Published:** 2021-12-29

**Authors:** Srividhya Srinivasan, Ramya Vengidesh, Anupama Ramachandran, Sadasiva Kadandale

**Affiliations:** 1 Conservative Dentistry and Endodontics, Chettinad Dental College and Research Institute, Chennai, IND

**Keywords:** triple antibiotic paste, open apex, obturation, immature permanent teeth, biodentine

## Abstract

Pulpal and periapical pathosis in an immature anterior tooth following traumatic injury is a frequent occurrence, and management of open apices in such cases poses a constant challenge to endodontists. This is due to the absence of apical constriction that would result in poor three-dimensional seal/adaptation of the obturated material within the canal system. Treatment of immature pulpless teeth with long-term calcium hydroxide may consequently weaken the dentin and increase the fracture susceptibility. Obturating the root canals completely with bioactive agents like Biodentine/mineral trioxide aggregate (MTA) has benefits like increased fracture resistance when compared to apexification. When used for obturation, Biodentine™, a new calcium silicate-based cement, performs superior to other Portland cement derivatives. This case report demonstrates a six-month follow-up result of an open apex and a periapical lesion involving maxillary right central incisor #11 with the Biodentine obturation that was previously subjected to triple antibiotic paste as an intra-canal medicament for three weeks. The satisfactory healing rate of tooth 11 with the reduction in the size of periapical radiolucency at the end of the six-month follow-up in this current article is highly considerable.

## Introduction

Dental traumatic injuries are most common in children, occurring between the age groups of 8 and 12 years. These injuries might lead to pulpal necrosis, which would cease the root formation, thereby resulting in an immature root apex [1]. Due to the open apices and weaker dentinal walls, the efficient management both from the restorative and endodontic perspective becomes quite cumbersome, which would often predispose to root fractures especially at the cervical region [2]. 

The apexification procedure is a viable choice of management in the case of immature permanent teeth with open apices. Though various materials have been suggested for apexification procedures, calcium hydroxide has been extensively used for apical barrier formation [3].

The management of pulpless immature teeth was earlier accomplished through the long-term (up to 18 months) use of calcium hydroxide to induce an apical barrier, but owing to its ability to debilitate/deteriorate the dentin in terms of its strength and due to a reduction in its fracture resistance, such usage is not actively preferred these days [1]. Several studies done in this regard have shown that apexification can form a hard tissue apical barrier, but the teeth might be more prone to cervical fracture due to thin dentin walls at the cementoenamel junction (CEJ) [4].

An alternative to calcium hydroxide would be silicate-based materials like mineral trioxide aggregate (MTA), Biodentine, etc., and these materials are found to be biocompatible, which could simulate biomineralization, thereby offering a superior seal and better bond strength [5]. 

Biodentine™, a bioceramic material commonly advertised nowadays as a "bioactive dentin substitute," possesses improved mechanical, physical, as well as handling properties comparable to those of MTA [6]. Due to their bioactive nature, these MTA and Biodentine have the ability to form a hydroxyapatite layer between the obturating materials and root dentin, which reinforces the effect of complete obturation with these materials [7]. In comparison to other calcium silicate-based cement-like MTAs, Biodentine offers three major advantages, which include superior mechanical properties, a faster setting time (12 minutes only), and better handling characteristics [4].

Thus, the current case report is to demonstrate the six-month follow-up of an open apex with a periapical pathology that was managed by using Biodentine as a core obturating material following the three weeks of triple antibiotic paste medication in the canal.

## Case presentation

**Figure 1:**
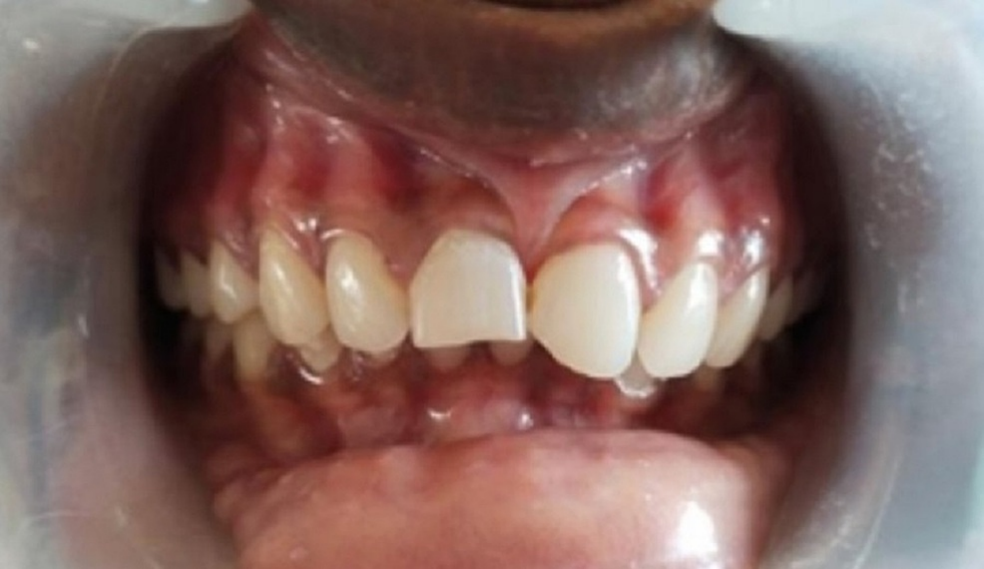
Pre-operative clinical photograph showing enamel and dentine fracture evident in teeth 11 and 21

A 17-year-old female patient reported to the Department of Conservative Dentistry and Endodontics with aesthetic concerns in her maxillary anterior region following a traumatic injury. While evaluating the history, the patient reported a dental trauma that had occurred nine years ago that resulted in a coronal tooth fracture of the right and left maxillary central incisors with no discoloration. The patient continued to show no symptoms of pain or dentinal hypersensitivity since the occurrence of the trauma, but there was an evident loss of enamel and dentin in teeth 11 and 21 (Figure 1).

**Figure 2:**
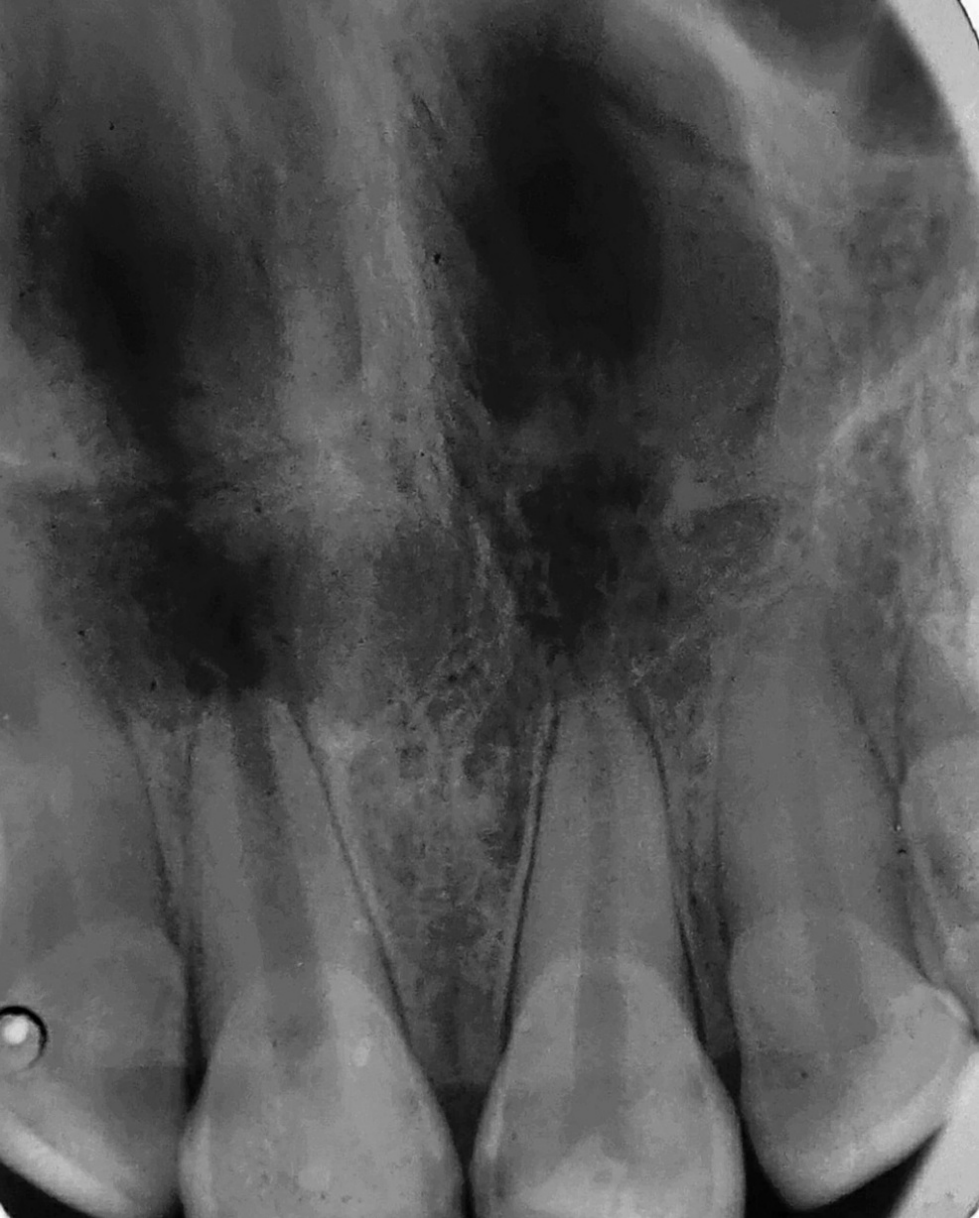
Intra-oral periapical radiograph revealing an open apex along with a periapical radiolucency of 1.5 cm diameter in tooth 11

Electrical pulp testing (Sybron Endo vitality scanner, 2006) revealed a positive response with the left maxillary central incisor but was negative with that of the right. Along with the electric pulp testing, an exploratory intraoral periapical radiograph was taken in relation to teeth 11 and 21. The radiograph revealed an open apex along with a periapical radiolucency of 1.5 cm in diameter present in regard to tooth 11 (Figure 2). The treatment plan was to place the triple antibiotic paste for three weeks to reduce the microbial load in the canal, followed by Biodentine obturation.

Access cavity preparation was done for tooth 11 under rubber dam isolation, and working length was determined by Ingle’s radiographic method and confirmed with an electronic apex locator. Shaping was done by scraping the canal walls with a size 50 K file to remove the irregularities in the walls. Then, the canals were irrigated copiously with 5.25% of sodium hypochlorite (Chemident, Delhi, India) and saline alternatively. This was followed by the preparation of triple antibiotic paste and its placement into the canal. The preparation involved T. metronidazole 400 mg + T. ciprofloxacin 500 mg + T. doxycycline 200 mg tablets whose sugar coating layer was scraped off with the help of a sterile No. 15 BP blade before the procedure. The tablets were then powdered, and saline was used as a vehicle to mix the antibiotic mixture, and finally the paste was obtained. This paste was then carried inside the canal using a paste carrier and a closed dressing was given using interim restorative material (Dentsply, intermediate restorative material [IRM]). The patient was then recalled after three weeks for the completion of Biodentine obturation. 

On the second visit, the tooth was again isolated with a rubber dam, and the triple antibiotic mixture was removed from the canal. The irrigation was then done with 5.25% of sodium hypochlorite and 17% EDTA (Prevest Denpro EDTA 17% solution). Biodentine™ (Septodont, St. Maur‑des‑Fosses, France) was manufactured in the form of a capsulated powder and a liquid twist cap bottle was used. The capsule containing the powder was gently tapped and then opened, which then received five drops of liquid from the twist cap bottle. The capsule was tightly shut and vibrated in an amalgamator for 30 seconds. The thick, creamy consistency obtained by the Biodentine can be handled for about six minutes during manipulation and takes another six minutes for the setting process. The material was carried to the orifice region with the help of an amalgam carrier.

**Figure 3:**
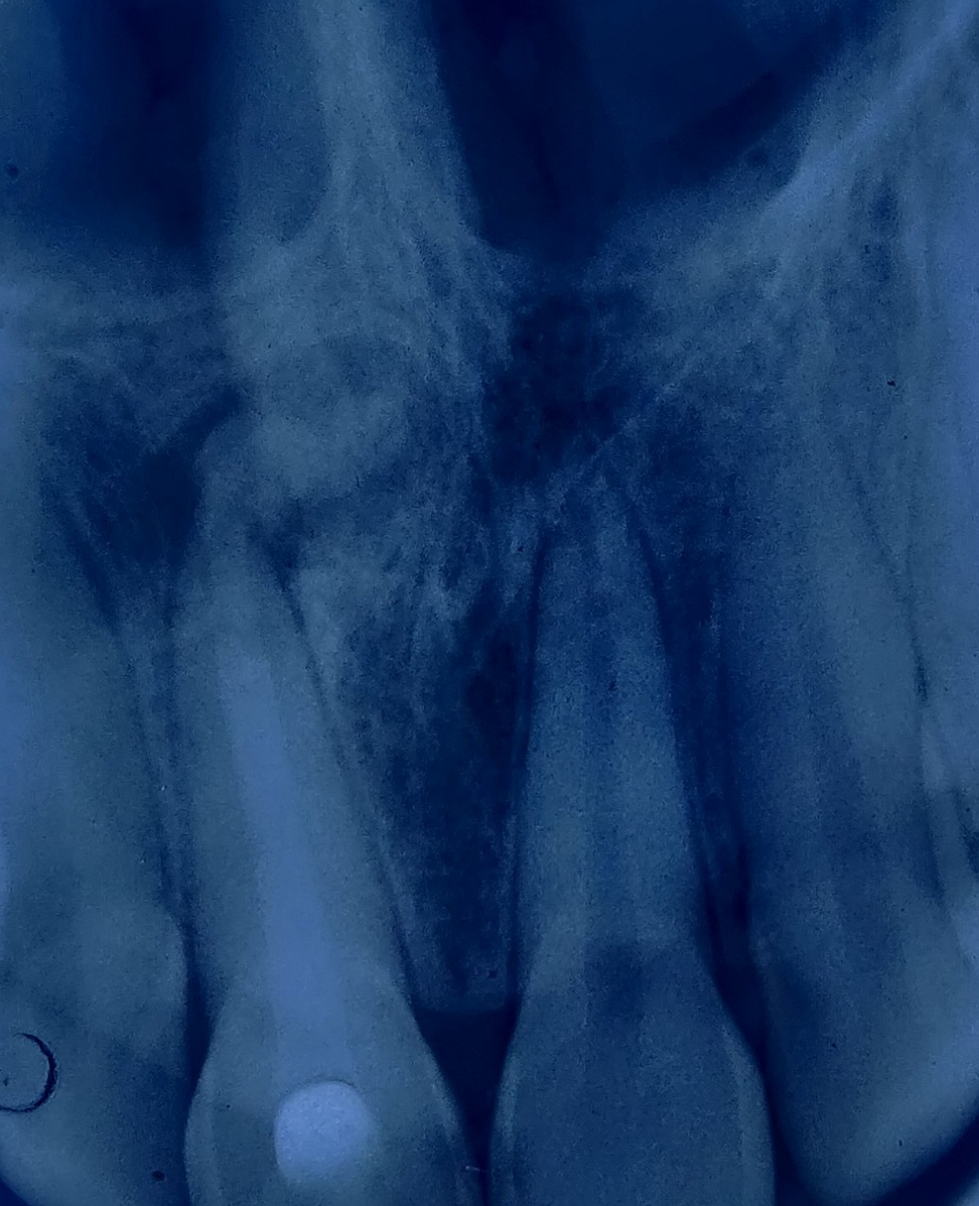
Post-operative intra oral periapical radiograph with access sealed with composite resin after a month

**Figure 4:**
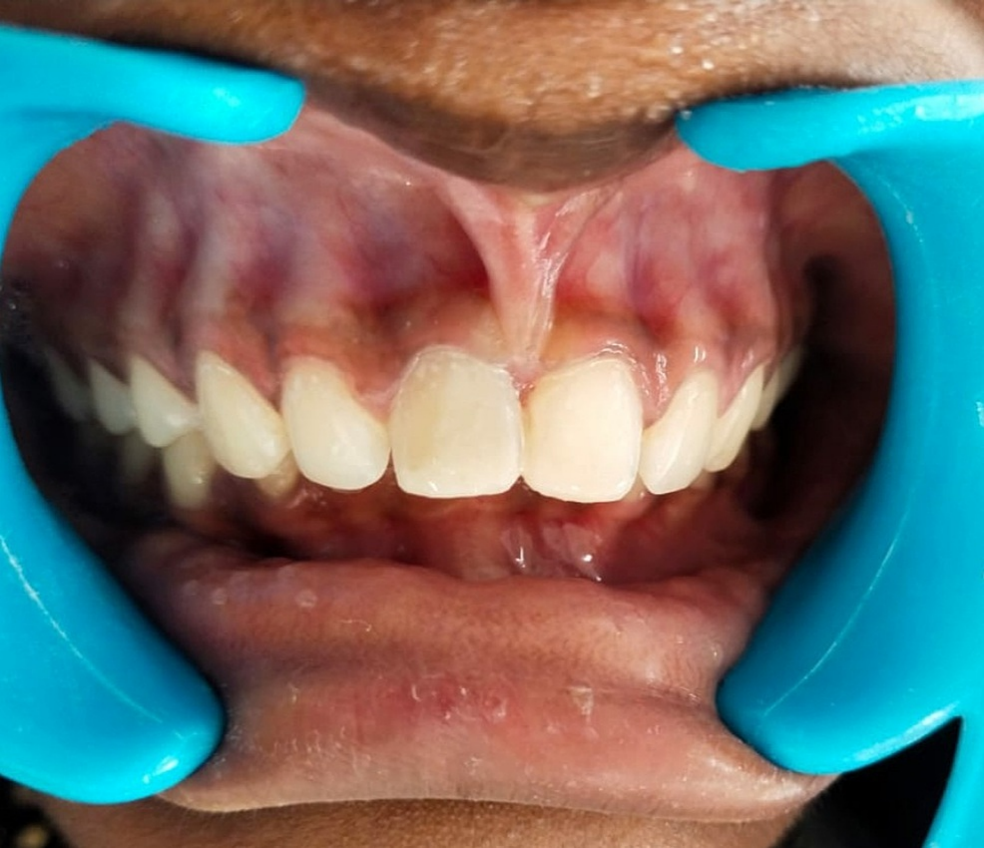
Post-operative clinical photograph with restored teeth 11 and 21 after a month

**Figure 5:**
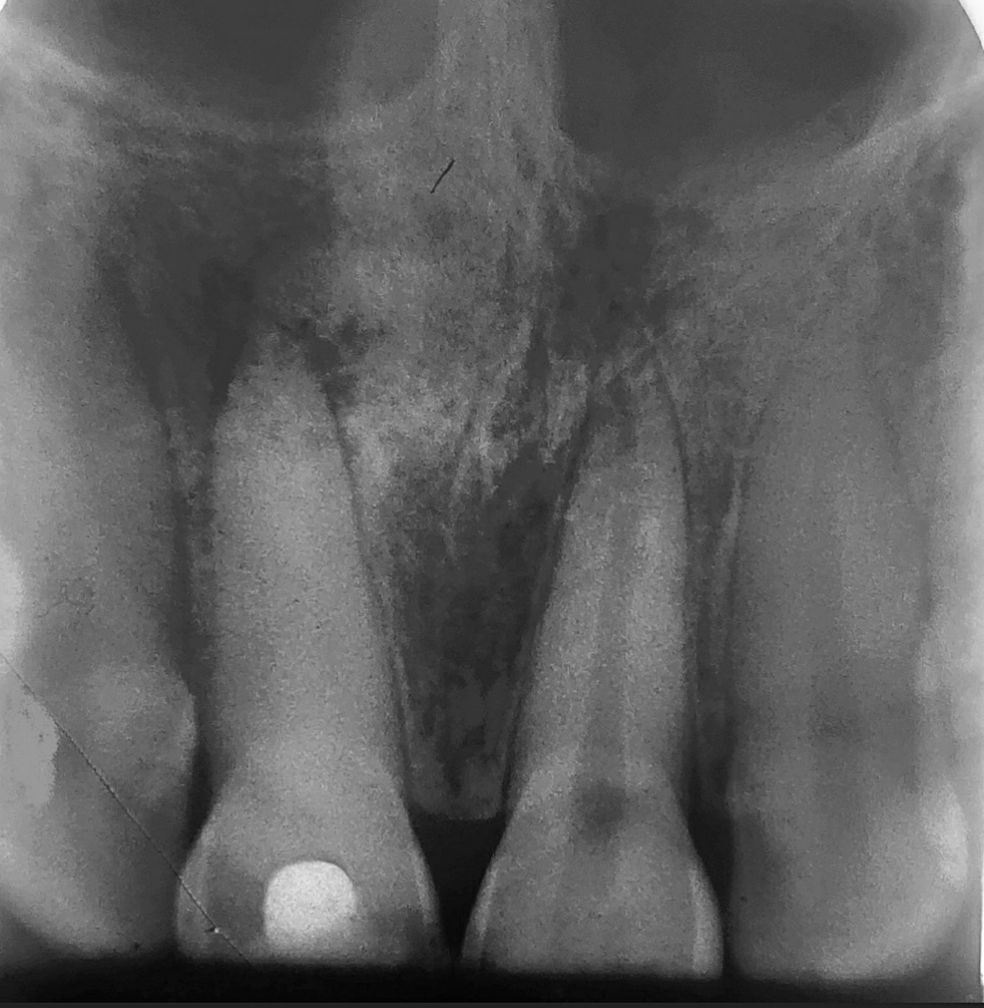
Post-operative intra oral periapical radiograph after six-month follow-up

After placing Biodentine into the canal, the material was carefully pushed towards the apical region (orthograde Biodentine obturation technique) with the help of a root canal plugger [(GDC root canal plugger (0.60 mm)]. The root canal was entirely obturated with Biodentine by placing it in several increments and then condensing with the help of a plugger. Post-obturation sealing of the canal orifice was done with glass ionomer cement followed by composite resin. The patient was recalled for a review after one, three, and six months. After a brief one-month period, the fracture extending up to the dentin was managed conservatively using direct composite restorations in teeth 11 and 21 (Figures 3-4). The history obtained from the patient and the clinical examination both revealed satisfactory healing after third- and sixth-month follow-up (Figure 5).

. 

## Discussion

The nature of fragile and thin dentinal walls in open apices means they are often fracture prone. The disinfection of canals and necrotic debris from the root canal system is mostly unachievable. The increased risk of overextension of materials into the periapex, along with the cleaning and shaping difficulties, all together result in offering a severe challenge to the clinician while managing the teeth with open apices.

The techniques used to obturate the immature teeth with the use of root canal obturating material without induction of apical closure include the placement of a customized gutta-percha cone or large gutta-percha along with the sealer at the apical region or gutta-percha placed short of the apex with the sealer. However, the above-mentioned management modalities do not offer an apical barrier. The other techniques intended to provide an apical barrier include placing calcium hydroxide to provide a mineralized apical barrier and placing biocompatible agents like dentinal chips against which the obturating material is placed. Traditionally, and until very recently, apexification was the procedure to manage the immature apex [8]. But the current case report demonstrates the procedure as well as the importance of obturating the canal with Biodentine due to its wide benefits.

The role played by intracanal medicament in the elimination of pathologic microbiota can never be underestimated, as the biomechanical/chemo-mechanical preparation alone will not render the root canal system microbe-free. Though elimination of microbes in case of persistent/large periapical pathosis has always been an arduous task for the clinicians, several antibiotic combinations especially the usage of Metronidazole, Minocycline, and ciprofloxacin [commonly termed as Triple antibiotic mixture] was found to be highly effective in eradicating the endodontic pathological microbiota both in-situ and in-vitro [9].

However, administration of a single antibiotic may not be effective in rendering the canal bacteria-free due to the varied microbial flora present in the canal system, typically including both aerobic and anaerobic species. To overcome these shortcomings, the Lesion Sterilization and Tissue Repair (LSTR) concept by the Cariology Research Unit of the Niigata University School of Dentistry was proposed [10]. This employs the usage of a triple antibiotic mixture of metronidazole, minocycline, and ciprofloxacin for disinfection [11].

Literature evidence from the past reports that usage of TAP has varied beneficial results for traumatized teeth presenting with periapical pathosis [12]. In their case report (2005), Ozan and Er et al. [13] concluded that the usage of combination antibiotic drugs in the triple antibiotic paste as an antimicrobial medicament is very predictable in resolving the large cyst-like lesions. The current case report records the usage of triple antibiotic paste for three weeks, after which the obturation with bioactive material, i.e., Biodentine, was carried out.

Following its application, Biodentine causes mineralization. By producing markers of odontoblasts and increasing transforming growth factor-beta1 (TGF-Beta1) release from pulpal cells, initial calcification occurs by forming osteodentine. Calcium hydroxide is generated during the cement setting process. Due to its high pH, calcium hydroxide produces irritation at the site of exposure. The zone of coagulative necrosis is thought to trigger precursor cell division and migration to the substrate surface, as well as addition and cyto-differentiation into odontoblast-like cells [14].

Biodentine stimulates odontoblasts to promote reactionary dentine apposition and cell differentiation to cause reparative dentin apposition [15,16]. It has an inhibitory effect on microorganisms due to its high alkalinity.

In general, the interradicular type of biofilm is found in the long-standing cases of periapical lesions [17]. Obturating the canals completely with bioactive agents like Biodentine and MTA not only possesses the advantages of cementum-like tissue formation, but also materials like MTA are potent antibacterial agents [18]. The mechanism involved in the formation of cementum-like tissues by calcium silicate cement includes the presence of calcium ions and Si-OH groups that deposit the apatite on to the root cement surface [19]. Also, the Hertwig root sheath regulates the differentiation of apically present periodontal ligament (PDL) cells that form the cementum-like tissue [20]. Further, the GP sealer interface present can shelter a huge number of tenacious gram-positive bacterial species and fungi. These microbes generally thrive between the root dentine and sealer/gutta-percha [21]. Moreover, it is very imperative to obtain a three-dimensional seal within the canal and avert bacterial penetration. To achieve this, the material used for obturation should adhere and adapt to the dentinal walls of the canal [22]. 

From the literature, it is evident that Biodentine possesses superior sealing ability and biocompatibility with the least cytotoxicity than other agents used for pulpal therapy [23]. From a study conducted by Kokate and Pawar et al. that compared the microleakage among MTA, Biodentine, and glass ionomer cement, it was concluded that Biodentine™ showed the least microleakage in comparison with other materials when used as a retrograde restorative material [24]. Another systematic review assessing the biocompatibility and sealing ability of Biodentine and MTA concluded that the favorable sealing ability exhibited by Biodentine along with its better biological characteristics suggest its efficient use as a retrograde restorative material clinically [25]. Malhotra and Hegde stated that the decreased particle size of Biodentine was claimed to be the reason for improved cavity surface wall and restorative adaptation [26]. Also, the reduction in the pore volume and porosity of Biodentine in comparison with MTA ensued in better sealing properties. Other contributing factors like faster rate of setting to avert the prolonged leakage and the ability to form the bio-mineralization tag, etc., are the noticeable properties present in Biodentine when compared to that of MTA [25].

Considering the importance of obturating the canal three-dimensionally with the better sealing ability and due to the many above-mentioned advantages of Biodentine over MTA, the obturation of the immature open apex in this case report was performed with Biodentine.

## Conclusions

Thus, trauma to the anterior teeth often results in an open apex and pulpal necrosis. Management of anterior teeth following trauma becomes a major concern and also a true challenge. Due to the high aesthetic demands of patients, immediate care of a high standard is always felt to be mandatory. Complete obturation of the root canal system and reinforcement of immature teeth with bioactive materials like Biodentine/MTA could be salutary. Thus, the material Biodentine can be advocated over MTA as an obturation material due to its favorable handling properties and rapid setting time.
